# *GPD1* and *ADH3* Natural Variants Underlie Glycerol Yield Differences in Wine Fermentation

**DOI:** 10.3389/fmicb.2018.01460

**Published:** 2018-07-03

**Authors:** Sebastián M. Tapia, Mara Cuevas, Valentina Abarca, Verónica Delgado, Vicente Rojas, Verónica García, Claire Brice, Claudio Martínez, Francisco Salinas, Luis F. Larrondo, Francisco A. Cubillos

**Affiliations:** ^1^Departamento de Biología, Facultad de Química y Biología, Universidad de Santiago de Chile, Santiago, Chile; ^2^Centro de Estudios en Ciencia y Tecnología de Alimentos, Universidad de Santiago de Chile, Santiago, Chile; ^3^Millennium Institute for Integrative Systems and Synthetic Biology, Santiago, Chile; ^4^Departamento de Genética Molecular y Microbiología, Facultad de Ciencias Biológicas, Pontificia Universidad Católica de Chile, Santiago, Chile; ^5^Departamento de Ciencia y Tecnología de los Alimentos, Facultad Tecnológica, Universidad de Santiago de Chile, Santiago, Chile

**Keywords:** wine, yeast, glycerol, alleles, *Saccharomyces*

## Abstract

Glycerol is one of the most important by-products of alcohol fermentation, and depending on its concentration it can contribute to wine flavor intensity and aroma volatility. Here, we evaluated the potential of utilizing the natural genetic variation of non-coding regions in budding yeast to identify allelic variants that could modulate glycerol phenotype during wine fermentation. For this we utilized four *Saccharomyces cerevisiae* strains (WE - Wine/European, SA – Sake, NA – North American, and WA – West African), which were previously profiled for genome-wide Allele Specific Expression (ASE) levels. The glycerol yields under Synthetic Wine Must (SWM) fermentations differed significantly between strains; WA produced the highest glycerol yields while SA produced the lowest yields. Subsequently, from our ASE database, we identified two candidate genes involved in alcoholic fermentation pathways, *ADH3* and *GPD1*, exhibiting significant expression differences between strains. A reciprocal hemizygosity assay demonstrated that hemizygotes expressing *GPD1^WA^*, *GPD1^SA^*, *ADH3^WA^* and *ADH3^SA^* alleles had significantly greater glycerol yields compared to *GPD1^WE^* and *ADH3^WE^*. We further analyzed the gene expression profiles for each *GPD1* variant under SWM, demonstrating that the expression of *GPD1^WE^* occurred earlier and was greater compared to the other alleles. This result indicates that the level, timing, and condition of expression differ between regulatory regions in the various genetic backgrounds. Furthermore, promoter allele swapping demonstrated that these allele expression patterns were transposable across genetic backgrounds; however, glycerol yields did not differ between wild type and modified strains, suggesting a strong *trans* effect on *GPD1* gene expression. In this line, Gpd1 protein levels in parental strains, particularly Gpd1p^WE^, did not necessarily correlate with gene expression differences, but rather with glycerol yield where low Gpd1p^WE^ levels were detected. This suggests that *GPD1^WE^* is influenced by recessive negative post-transcriptional regulation which is absent in the other genetic backgrounds. This dissection of regulatory mechanisms in *GPD1* allelic variants demonstrates the potential to exploit natural alleles to improve glycerol production in wine fermentation and highlights the difficulties of trait improvement due to alternative *trans*-regulation and gene-gene interactions in the different genetic background.

## Introduction

Glycerol production is one of the most important by-products generated during alcohol fermentation. Depending on the quantity and wine type, glycerol can contribute to wine flavor intensity and impact aroma volatility (Gawel et al., 2007; Marchal et al., 2011; Zhao et al., 2015). In budding yeast, glycerol is synthesized by the reduction of dihydroxyacetone phosphate followed by dephosphorylation catalyzed by glycerol-3-phosphate dehydrogenase (*GPD1*) and glycerol-3-phosphatase (*GPP1*) (Albertyn et al., 1994). Genetic modification has been used to engineer yeast that produces more glycerol (Steensels et al., 2014). Despite this, the application of genetically modified organisms (GMOs) in the industry is restricted by the lack of policies that regulate their use and by negative public perception (Steensels et al., 2014). This has inspired the development of alternative strategies for the generation of new strains, such as experimental evolution (Steensels et al., 2014; Tilloy et al., 2014). For example, the wine strain EC1118 has been genetically improved to produce more glycerol through constant exposure to osmotic stress (Tilloy et al., 2014). Nevertheless, the use of experimental evolution to obtain specific phenotypes is time-consuming, and undesirable mutations can complicate industrial applications. Thus, non-invasive nor mutagenic strategies represent an alternative where variants of interest are selected from standing natural genetic variation (Cubillos, 2016). *S. cerevisiae* strains are genotypically and phenotypically highly variable, and thus are an ideal model for studying trait improvement (Thompson and Cubillos, 2017; Peter et al., 2018).

Natural and commercial *S. cerevisiae* isolates differ largely in a series of traits (Crepin et al., 2012; Salinas et al., 2016; Cubillos et al., 2017). In this context, it has been reported that depending on the genetic background, isolates can yield different concentrations of acetic acid, glycerol, ethanol, and other secondary metabolites (Salinas et al., 2012). Efforts aimed at deciphering the genetic basis underlying some of these phenotypic differences in isolate types have demonstrated the existence of a wide set of quantitative trait loci (QTLs), for example: ethanol production (Katou et al., 2009; Pais et al., 2013), ethanol tolerance (Swinnen et al., 2012), glycerol production (Hubmann et al., 2013b), asparagine assimilation (Marullo et al., 2007), low temperature fermentation (Garcia-Rios et al., 2017), and nitrogen assimilation (Brice et al., 2014, 2018; Cubillos et al., 2017). In most of these cases, QTLs are down to non-synonymous changes which significantly impact protein structure and gene function. For example, a series of aminoacidic changes in *SSK1*, *GPD1*, *HOT1*, and *SMP1* genes have been found as responsible for low glycerol and high ethanol yield differences between CBS6412 and Ethanol Red strains (Hubmann et al., 2013a,b). Yet, the molecular mechanisms and the effect of these polymorphisms upon protein activity and stability are unknown.

Although, these regions explain a substantial fraction of the natural phenotypic variation between individuals, a wide set of variants across eukaryotes are located within non-coding regions and finely modulate gene expression and ultimately phenotypes (Wray, 2007). In this context, non-coding regions have been less explored in yeast and could be useful for genetic breeding and industrial applications via the modulation of gene regulation and expression (Thompson and Cubillos, 2017). Previous expression profiles of *S. cerevisiae* isolates obtained from different ecological niches have demonstrated that the genetic control of expression is well-defined (Fay et al., 2004; Kvitek et al., 2008; Ehrenreich et al., 2009; Zhu et al., 2009; Fraser et al., 2010; Cubillos et al., 2012). Additionally, budding yeast can be easily manipulated at the molecular level and represents a great model for genetic improvement and for understanding the consequences of mutations within coding and regulatory regions (Salinas et al., 2016). For example, early QTL mapping on sporulation efficiency between two North-American isolates has validated the role of non-coding regions on natural variation in yeast by showing the effects of a single nucleotide deletion upstream of *RME1* (Gerke et al., 2009). In this context, we have previously demonstrated how widespread Allele Specific Expression (ASE) is across four *S. cerevisiae* isolates representative of different lineages of the species. (Salinas et al., 2016). Interestingly, estimates of the aspartic acid and glutamic acid consumption in the wine fermentation must of two yeast strains from different geographic origins have demonstrated that polymorphisms in both portions (coding and regulatory) of the *ASN1* gene, are partly responsible for nitrogen assimilation differences between genetic backgrounds (Salinas et al., 2016). Moreover, this study provided a catalog of *cis*-variants between strains that directly influence allelic expression and which can be used as tools for the dissection of other phenotypes.

In this study, we utilize the existing standing genetic variation in yeast within non-coding regions to identify natural allelic variants for genes part of the alcoholic fermentation pathways that could impact glycerol production under synthetic wine must (SWM) conditions. For this, we searched our ASE database for genes involved in fermentation, such as alcohol dehydrogenases and in glycerol biosynthesis. From this, we studied two candidate genes, *ADH3* and *GPD1*, with differently expressed alleles between strains. Through reciprocal hemizygosity, allele swapping, along with transcriptional and co-translational profiling across strains, we demonstrate that *ADH3* and *GPD1* allelic variants modulate glycerol yield and could be used as natural sources for genetic improvement and gene expression fine tuning.

## Materials and Methods

### Yeast Strains and Culture Media

The haploid strains Y12 (referred to as Sake, ‘SA’, *Mat alpha ho::HygMX*, *ura3::KanMX*), YPS128 (referred to as North American, ‘NA’, *Mat alpha ho::HygMX*, *ura3::KanMX*), DBVPG6044 (referred to as West African, ‘WA’, *Mat alpha ho::HygMX*, *ura3::KanMX*) and DBVPG6765 (referred to as Wine/European, ‘WE’, *Mat a, ho::HygMX*, *ura3::KanMX*) together with F1 hybrids (WE x SA, WE x NA, and WE x WA crosses) utilized in this study have been previously described (Cubillos et al., 2009, 2011). Before every experiment, strains were recovered from frozen glycerol stocks in rich yeast peptone dextrose (YPD) agar media and grown overnight at 28°C.

### Fermentation in Synthetic Wine Must (SWM) MS300 and HPLC Analysis

Fermentations were carried out in at least three biological replicates depending on the experiment. Fermentations were conducted using SWM supplemented with 300 mgN/L) (MS300, hereafter referred to as SWM) and 270 g/L of total sugar (glucose and fructose and prepared as previously reported (Rossignol et al., 2003) (Jara et al., 2014). For each experiment, the strains were initially grown with constant agitation in 10 mL of SWM for 16 h at 25°C. Following this, 12 mL of fresh SWM were inoculated to a final concentration of 1x10^6^ cells/mL of yeast (in 15 mL conical tubes) and incubated at 25°C with no agitation. Fermentations were weighed every day to calculate the CO_2_ output. The fermentations were maintained until the daily CO_2_ lost represented less than 10% of the accumulated CO_2_ lost. At the end of the fermentation, the fermented SWMs were centrifuged at 9000 ×*g* for 10 min and the supernatant was collected. From this, the concentration of extracellular metabolites was determined using HPLC. Specifically, 20 μL of filtered must were injected in a Shimadzu Prominence HPLC (Shimadzu, United States) with a Bio-Rad HPX –87H column (Nissen et al., 1997). In this way, the concentrations of glucose, fructose, trehalose, acetic acid, succinic acid, malic acid, ethanol, and glycerol was estimated (results found in **Supplementary Tables S2**, **S4**). Ethanol yield was estimated converting %v/v to g/L utilizing the ethanol density and then dividing by total sugar consumption. Similarly, glycerol yield was estimated by dividing the observed glycerol levels (g/L) by the total amount of sugar consumed.

### Reciprocal Hemizygosity Assay

Reciprocal hemizygotes of the *ADH3* and *GPD1* candidate genes were generated as previously described (Cubillos et al., 2013; Jara et al., 2014; Salinas et al., 2016). Briefly, the *URA3* gene previously deleted in the haploid parental strains (Cubillos et al., 2009) was used as a selectable marker for the deletion of each target gene. The haploid versions of the parental strains also contained opposite antibiotic markers in the *HO* locus (Hygromycin B for “Mat *a*” strains and Nourseothricin for “*alpha*” strains), which allowed us to cross the haploid mutant parental strains and construct all possible combinations of single deletions. Thus, mutated parental strains were crossed to generate the reciprocal hemizygote strains, selecting the diploid hybrids in antibiotic plates (300 ug/mL of Hygromycin B and 100 ug/mL of Nourseothricin). Finally, diploids were confirmed by *MAT* locus PCR (Huxley et al., 1990). Primers are listed in **Supplementary Table S1**.

### Luciferase Expression Assay (Cloning and Phenotyping)

The *GPD1* genetic constructs carrying the destabilized version of the firefly luciferase reporter gene under the control of the different regulatory allelic variants were assembled using yeast recombinational cloning as previously described (Salinas et al., 2016). Briefly, 700 bp upstream of the ATG start codon (regulatory region) and the firefly luciferase gene (Rienzo et al., 2012) were amplified by PCR using Phusion Flash High-Fidelity PCR Master Mix (Thermo scientific, United States). In addition, the Hygromycin HphMx antibiotic resistance gene was amplified by PCR and was included in the genetic constructs. Overall, the overlap between PCR products was 50 bp and were co-transformed with the linear plasmid pRS426 in the yeast lab strain BY4741 (*MATa*, *his3Δ1*, *leu2Δ0*, *LYS2*, *met15Δ0*, *ura3Δ0*). The circular plasmids generated in yeast were transferred to an *E. coli* DH5α strain and confirmed by colony PCR using standard conditions. At least three positives colonies containing the regulatory region, the luciferase gene, and the HphMx cassette were selected for plasmid isolation and sequencing. The sequence identity of the regulatory regions was confirmed using the SGRP2 BLAST database service (Bergstrom et al., 2014). Finally, the parental strains were transformed with the complete genetic constructs, which were amplified by PCR using a Phusion Flash High-Fidelity PCR Master Mix (Thermo scientific, United States). For the latter, 70 bp primers were utilized, which guided direct homologous recombination at the target locus, allowing for the integration of the genetic constructions in the genome. The positive yeast colonies were analyzed by colony PCR with standard conditions.

The use of a destabilized firefly luciferase (Rienzo et al., 2012) allowed quantifying expression of the targeted genes in real-time and, to avoid the effects of copy number and genetic context on gene expression, the genetic constructs were integrated into the original *GPD1* locus, maintaining the genetic context. Additionally, reciprocal hemizygotes were generated with these constructions as previously described. Strains carrying the firefly luciferase constructs were analyzed for luciferase expression using a Cytation3 microplate reader (Biotek, United States). Briefly, the strains were pre-grown in YNB (Yeast nitrogen base, supplemented with 2% glucose) and SWM overnight. The cultures were then diluted 1/100 to inoculate a 96 well plate with 200 uL of fresh culture media containing 0.1 mM of luciferin. The *in vivo* OD_600_nm and the luminescence intensity of the cell cultures were monitored every 10 min. All the experiments were performed using, at least, three biological replicates.

### Allele Swapping

Promoter allele swaps were carried out as previously described (Salinas et al., 2016). Briefly, genetic constructs carrying the regulatory regions of *GPD1* and *ADH3* (700 bp upstream of the ATG start codon) plus the HphMx cassette in the reverse direction (*HphMxRv-P_GPD1_*) were assembled using yeast recombinational cloning. See details above for full descriptions. Initially, we used the WE strain as a receiver of the promoters coming from the NA, SA, and WA strains. For this, the regulatory region of the target gene was deleted in the WE strain using *URA3* gene as a selectable marker. Then, the construct containing the promoter of interest was amplified by PCR and used for transformation and direct recombination with the regulatory region. The final strains were confirmed by standard colony PCR and sequencing. Furthermore, we used the NA, SA and WA strains as receivers of the promoter coming from the WE strain following the same procedure.

Additionally, we used the strains carrying the promoter swaps as recipients of the luciferase reporter gene for direct quantification of gene expression in living cells. Again, see above for the full description of the methods. The construct containing the destabilized version of the luciferase gene plus the *URA3* selectable marker (*Luc-URA3*) was amplified by PCR and used for *GPD1* transformation of the strains carrying the promoter swaps. The final strains were confirmed by colony PCR using standard conditions.

### Gpd1p Tagging With mCherry

The fusion of the Gpd1 protein with mCherry was carried out using one step PCR and recombination with the 3′end of the *GPD1* ORF, which corresponds to the C-terminal of the Gpd1 protein. This allowed us to remove the stop codon of the *GPD1* gene and fuse its ORF with the mCherry coding sequence (DeLuna et al., 2010). In this way, we generated a construct containing the mCherry sequence plus the hygromycin cassette (mCherry-HphMx). This allowed us to directly tag the *GPD1* ORF and perform selection by hygromycin in each yeast strain. The mCherry-HphMx construct was assembled in a pRS426 plasmid using the above described yeast recombinational cloning method (Oldenburg et al., 1997). The yeast strains carrying the mCherry-HphMx cassette were confirmed using standard yeast colony PCR.

The yeast strains were analyzed in microcultivation with a Cytation 3 microplate reader, which allowed for the dual measurement of OD_600_ and fluorescence of the cell cultures over time. Briefly, the yeast strains were grown overnight in a 96 well plate with 200 uL of YNB or SWM medium. 10 uL of these cultures were used to inoculate a new black 96 well plate containing 300 uL (30-fold dilution) of fresh media. The OD_600_ and the fluorescence were measured every 30 min using 587 nm of excitation and targeting emission wavelengths of 620 nm with a gain of 100 units.

### *GPD1* Sequence Analysis

*GPD1* sequences were obtained from the SGRP2 database (Bergstrom et al., 2014) and regulatory regions together with ORF sequences were compared using Geneious 8.1.5. Transcription factor binding sites were predicted utilizing YeTFaSCo: Yeast Transcription Factor Specificity Compendium (de Boer and Hughes, 2012). The Codon Adaptation Index (CAI) for each allele variant was estimated using the CAIcal server (Puigbo et al., 2008) with default settings and utilizing the standard *Saccharomyces cerevisiae* genome codons usage from the Codon Usage Database (Nakamura et al., 2000).

### Data Analysis

The significance of all comparisons was made through non-parametric test depending on whether two groups or multiples groups were compared. Fermentation metabolites results obtained from HPLC were compared across strains utilizing a non-parametric Kruskal-Wallis test and Dunn’s Multiple Test Comparison. Similarly, significance in metabolites levels between reciprocal hemizygotes were assessed utilizing a non-parametric Mann Whitney test. Gene expression and protein levels across the four parental strains were evaluated using a Friedman test and Dunn’s Multiple Test Comparison. Luciferase expression and glycerol yield was estimated utilizing a Spearman rank correlation test. Finally, gene expression and protein levels across hemizygotes were compared using a Wilcoxon signed rank test. All analyses were performed utilizing GraphPad Prism Software 5.2. In all cases *p*-values < 0.05 were considered as significant.

## Results

### Glycerol Production Differs Among *S. cerevisiae* Strains

Through the utilization of the ASE database, we aimed to identify natural allelic variants impacting glycerol production yields when grown in SWM. Thus, we initially characterized the fermentation profiles and ability to produce a series of metabolites in four strains grown in SWM. We estimated the concentrations of glucose, fructose, trehalose, acetic acid, succinic acid, malic acid, ethanol and glycerol after 21 days of micro-fermentations experiments.

**FIGURE 1:**
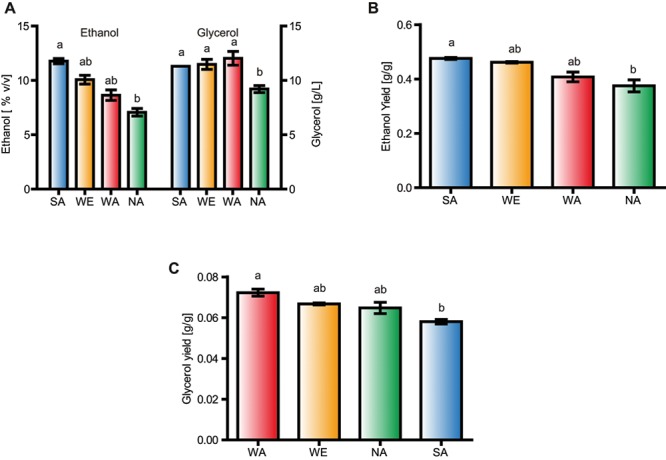
Glycerol production in *S. cerevisiae* strains. **(A)** Glycerol and ethanol levels; **(B)** Ethanol yield and **(C)** glycerol yield in Sake (SA), West African (WA), Wine/European (WE), and NA (North American strains) after fermentation in synthetic wine must. Different letters indicate significant differences between genotypes.

Ethanol and glycerol significantly differed among some isolates (**Supplementary Table S2** and **Figure 1A**, *p*-value < 0.05, and Kruskal-Wallis test). For example, the SA strain showed greater ethanol production than NA (**Figure 1A**). Conversely, the WA and the NA strain showed significant differences for glycerol production, the latter producing lower levels of glycerol (**Figure 1A**, *p*-value < 0.05, and Kruskal-Wallis test). Since we found relatively high amounts of residual sugars in our fermentations, we estimated yields to accurately measure the quantity of sugar transformed into ethanol and glycerol, respectively (**Figures 1B,C**). From this we observed that the WA strain (a non-domesticated strain) yielded significantly more glycerol than the SA strain (*p*-value < 0.05, Kruskal-Wallis test), in agreement with their glycerol and ethanol production levels, respectively. Thus, the WA strain produced the highest glycerol yields (**Figure 1C**), while the WE isolate, considered a domesticated strain, did not produce greater ethanol nor glycerol levels/yields compared to WA, demonstrating that other non-domesticated genetic backgrounds may represent potential sources of allelic variants that can be used to boost glycerol production in wine fermentation.

### Reciprocal Hemizigosity Assay (RHA) Validates Glycerol Yield Differences Among *ADH3* and *GPD1* Allelic Variants

**FIGURE 2:**
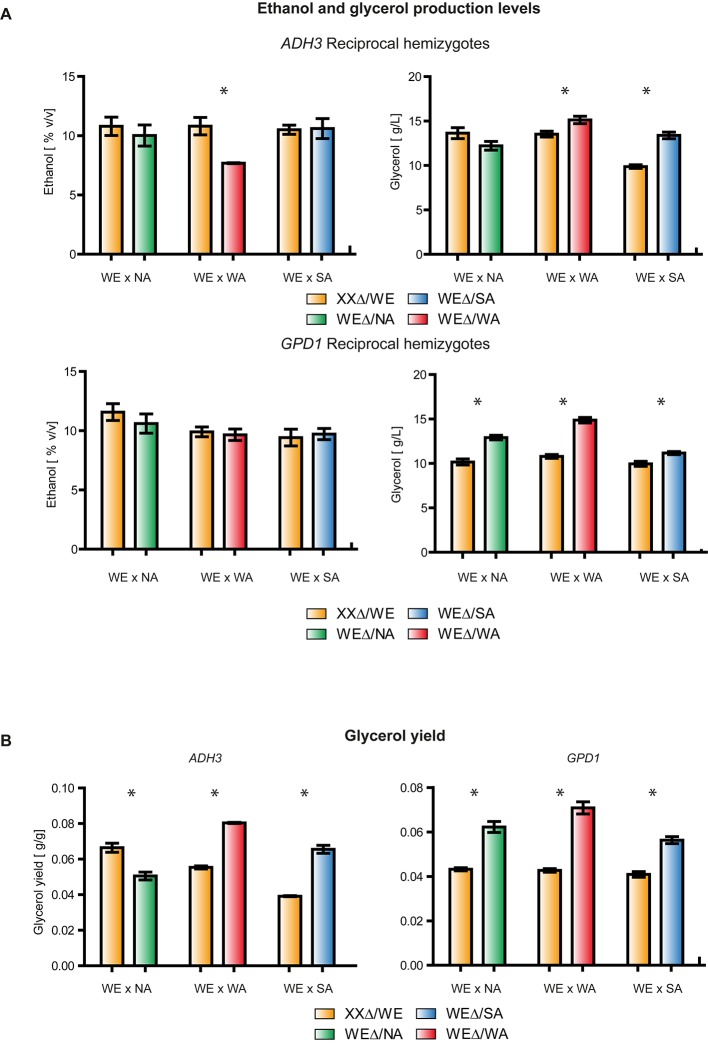
Reciprocal hemizygosity assay for *GPD1* and *ADH3*. **(A)** Ethanol (%v/v) and Glycerol (g/L) levels in WE × NA, WE × SA, and WE × WA reciprocal hemizygotes. The Glycerol yield for the same strains is shown in **(B)**. XX denotes either the NA, WA, or SA genotype depending on the bar. ^∗^*p*-value < 0.05.

In order to identify allelic variants that could influence glycerol yield, we utilized the ASE database (Salinas et al., 2016) to find ethanol/glycerol biosynthesis alleles with divergent expression. Additionally, since our focus was the wine fermentation environment, we only selected genes in which the WE allele was differently expressed (**Supplementary Table S3**). Here, we found two genes, *ADH3* and *GPD1*, which encode for an alcohol dehydrogenase III and a glycerol-3-phosphate dehydrogenase, respectively (Young and Pilgrim, 1985; Albertyn et al., 1994; de Smidt et al., 2008). Subsequently, to estimate the relative contribution of each genetic variant to ethanol and glycerol yields, we performed a functional analysis to compare the reciprocal hemizygotes derived from the three WE F1 hybrids. For both genes, we observed that hemizygotes carrying a WE variant produced substantially lower glycerol levels (except for *ADH3* - NA × WEΔ, *p*-value < 0.05, Mann Whitney test) than hemizygotes carrying the WA and SA allelic variants (**Figure 2A**). For example, *ADH3^WA^* (*ADH3* hemizygote carrying the WA variant) and *ADH3^WE^* hemizygotes produced 15.1 ± 0.4 g/L and 13.5 ± 0.6 g/L of glycerol, respectively. Similarly, this pattern was also observed for glycerol yields, where differences were maximized when sugar consumption was considered (**Figure 2B**). For example, the *GPD1^WA^* hemizygote yielded 66% more glycerol than the *GPD1^WE^* hemizygote. These results agree with the glycerol levels produced by the WA and WE parental strains, but not with SA strain, suggesting an antagonistic effect of the SA alleles. Interestingly, none of the hemizygotes with significantly greater glycerol yields had lower ethanol yields. Instead, WE hemizygotes produced increased yields of other metabolites including succinic acid and malic acid depending on the hybrid (**Supplementary Table S4**). This suggests quantitative differences in carbon molecules fluxes among strains. Overall, these results indicate that *ADH3* and *GPD1* allelic variants from the WA and SA strains could be used to maximize glycerol yields in wine fermentation; however, we found no evidence that selection of these alleles would affect ethanol production.

### Transcriptional Profiling Demonstrates That *GPD1* Expression Levels Are G x E Dependent

**FIGURE 3:**
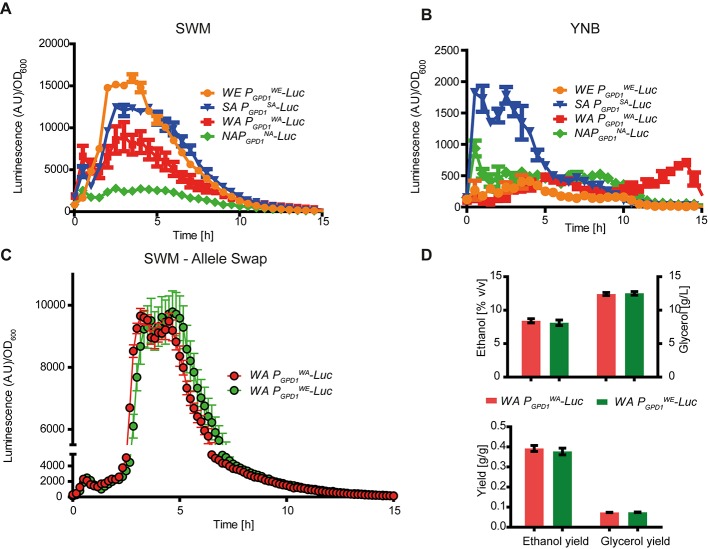
*GPD1* gene expression profile. Luciferase reporter gene expression assay for *GPD1* promoters in their native genetic backgrounds grown in **(A)** synthetic wine must and **(B)** yeast nitrogen base (YNB). **(C)** Allele swap in the WA strain with a luciferase reporter gene controlled by the wild type promoter (red) and the WE promoter (green). **(D)** Metabolite levels in the allele swap assay.

We obtained expression profiles for each allelic variant to determine how allelic differences in regulatory regions affected expression levels, timing, and were condition dependent. For this, we focused on *GPD1* since this gene was involved in the greatest glycerol yield differences among hemizygotes. We generated transcriptional fusions in all strains by inserting a destabilized luciferase reporter gene immediately downstream of the regulatory region and replacing the original *GPD1* locus (Salinas et al., 2016). Firstly, the luciferase expression levels were obtained for all parental strains under micro-cultivation conditions in YNB and in SWM to evaluate the strength of the promoters in these two scenarios. From the transcriptional expression profiling, assay we found differences in expression between strains and environments, clearly indicating a G × E interaction (**Figures 3A,B** and **Supplementary Figure S1**). For example, the luminescence of *P_GPD1_^WE^-Luc* (the WE *GPD1* promoter controlling luciferase gene expression) was lower when the strain was cultivated in laboratory media than when cultivated in SWM (*p*-value < 0.05, Friedman test). Overall, the expression of *P_GPD1_^WE^-Luc* under SWM was highest among all strains (**Figure 3A**, *p*-value < 0.05, Friedman test). Interestingly, when cultivated in SWM each strain had a unique *GPD1* expression profile with expression levels clearly increasing among strains, WE > SA = WA > NA (*p*-value < 0.05, Friedman test). Conversely, the results differed when the strains were cultivated in YNB media. Here, *P_GPD1_^SA^-Luc* exhibited the strongest luminescence (**Figure 3B**, *p*-value < 0.05, Friedman test). It is worth noting that the luciferase expression levels were ∼10 times higher when strains were cultivated in SWM than when cultivated in YNB. These results demonstrate that the expression of *GPD1* is highly induced under fermentative conditions and its strength is dependent on the promoter allelic variant, yet the role of *cis*- and/or *trans* regulation is uncertain. Interestingly, the parental strain expression profiles did not correlate with the glycerol yields previously estimated (**Figure 1** and **Supplementary Table S2**, *p*-value = 0.2 Spearman rank correlation).

In order to evaluate the role of the *cis*-regulatory region and whether the *P_GPD1_^WE^* could sufficiently increase expression levels and thus modify glycerol yield in other genetic backgrounds, we performed an allele swap. Immediately upstream of the luciferase reporter, we replaced the native *GPD1^WA^* regulatory region with *P_GPD1_^WE^* in the WA strain (700 bp upstream of the ORF). Microcultivation in SWM revealed that the WA strain carrying the *P_GPD1_^WE^* variant had greater expression than WA strains with the native promoter (*p*-value < 0.001, Wilcoxon signed rank test). Expression levels reached a maximum discrepancy of 18% around 5 h of cultivation (**Figure 3C**) and significant differences were found throughout the cultivation period (*p*-value < 0.001, Wilcoxon signed rank test). This demonstrates the role of *cis*- regions and the potential to increase *GPD1* expression in foreign genetic backgrounds. We next evaluated the impact of a promoter swap on glycerol yield. *P_GPD1_^WE^* was introduced in the WA strain controlling the expression of *GPD1^WA^* ORF. Fermentation was carried out for 21 days in SWM, and glycerol together with ethanol yields were estimated. No significant differences were found as raw metabolite levels and yields were similar between strains (**Figure 3D**), suggesting that the promoter itself is not sufficient to increase glycerol yield or that we did not have the experimental power to detect minor glycerol yield differences due to experimental noise.

**FIGURE 4:**
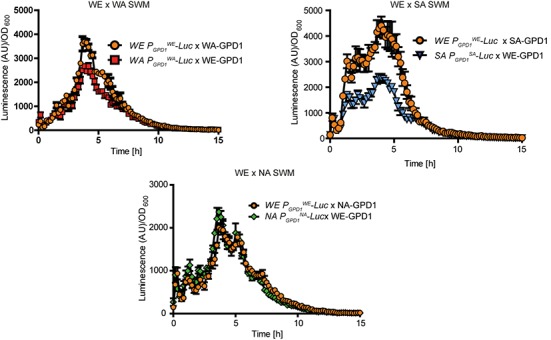
Luciferase reporter assay in *GPD1* reciprocal hemizygotes. Luminescence levels in *GPD1* WE × WA, WE × SA, and WE × NA hemizygotes in micro cultivation in SWM.

Subsequently, to evaluate the influence of all promoters on gene expression and to avoid polymorphic *trans*-effects that could modulate mRNA levels, we proceeded to examine expression levels in F1 reciprocal hemizygotes. Interestingly, we observed greater expression in the strains with the WE promoter controlling the reporter gene (*p*-value < 0.001, Wilcoxon signed rank test, **Figure 4** and **Supplementary Figure S2**). Specifically, expression was higher in the *P_GPD1_^WE^-Luc* x WA*-GPD1* (WE x WA F1 hybrid with the WE *GPD1* promoter controlling luciferase expression) and *P_GPD1_^WE^-Luc* x SA*-GPD1* hemizygotes (WE x SA F1 hybrid with the WE *GPD1* promoter controlling luciferase expression gene) compared to the *P_GPD1_^WA^-Luc* x WE*-GPD1* and *P_GPD1_^SA^-Luc* x WE*-GPD1* hemizygotes (**Figure 4**). No significant differences in expression were found for the WE x NA hemizygotes.

To identify putative transcription factor binding sites that could modulate *GPD1* gene expression, we analyzed the *cis*-regulatory region (up to 200 bp upstream of the ATG start site). This was done via sequence alignment and by predicting binding sites utilizing the YeTFaSCo database. (de Boer and Hughes, 2012). Three SNPs are found between the four strains, and two of these were exclusively present in the WE strain. The closest polymorphism to the ATG start site corresponded to a deletion of two nucleotides in the West African and North American strains (*del-32CC*). Nevertheless, this deletion does not yield polymorphic binding sites for this region, and the same transcription factors would bind in all strains. The other two polymorphisms upstream of *del-32CC* encode a thymine instead of a cytosine (*T-180C*) around nucleotide -180 (from the ATG site) and a cytosine instead of a guanine (*C-202G*) around -202 in the WE background. The last polymorphism could potentially influence allele specific binding and could alter Crz1 binding, which is a transcription factor associated with the response to ethanol stress (Araki et al., 2009).

Overall, these results suggest the presence of powerful *cis*-factors that increase *GPD1^WE^* levels independently of the genetic background. This being said, the lower glycerol yields in WE could be explained by other regulatory mechanisms (negative post-transcriptional regulation, post-translational modifications) or reduced Gpd1^WE^p activity.

### Gpd1-mCherry Fusions Suggest Negative Post-transcriptional Regulation on Gpd1p^WE^

**FIGURE 5:**
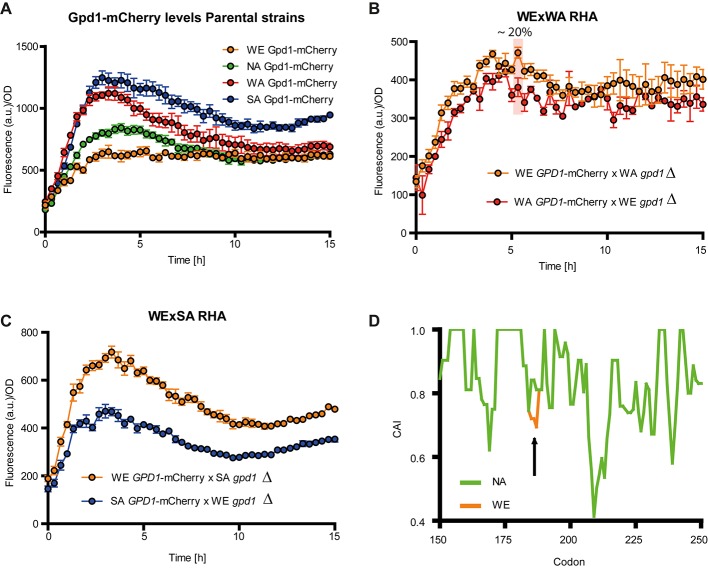
Gpd1p levels in *S. cerevisiae* strains. Gpd1-mCherry levels in **(A)** parental strains, **(B)** WE × WA reciprocal hemizygotes, and **(C)** WE × SA reciprocal hemizygotes in micro cultivation in SWM. **(D)** Codon Adaptation Index (CAI) in NA, WA or SA strains (green) respect to the WE strain for *GPD1* between codons 150 and 250. The arrow indicates the codon for which a CAI difference was found between *GPD1^NA^*, *GPD^WA^* and *GPD1^SA^* versus *GPD1^WE^*.

Since a negative correlation was found between *GPD1* expression levels and glycerol yield in the WE strain, we sought to quantify Gpd1 protein levels as a means to detect putative post-transcriptional regulation. For this, we generated a Gpd1p-mCherry fusion in all parental strains by genetically linking mCherry to the Gpd1p C-terminal. Protein levels were estimated in micro-cultivation in SWM. We found significant differences between all strains (*p*-value < 0.05, Friedman test). During the first hours of incubation, the mCherry fluorescence was strong in the SA and WA strains, whereas the signals in the WE and NA strains were low (**Figure 5A** and **Supplementary Figure S3**). Interestingly, the mCherry fluorescence of the SA and WA strains was two times higher than the WE strain. This indicates that the WE strain produces significantly less Gpd1p, which contrasts with the increased expression of *GPD1* in this strain. Overall, this suggests that post-transcriptional modifications affect protein levels and influence glycerol yields in all strains.

Subsequently, in order to evaluate the role of a dominant or recessive *trans*-factor we quantified protein fluorescence in the WE × WA and WE × SA hemizygotes (**Supplementary Figure S3**). We detected differences in the fluorescence among genotypes; the fluorescence of hemizygotes carrying Gpd1p^WA^ was up to 20% (at 5 h’ time point) lower than that of hemizygotes with Gpd1p^WE^. Overall, the fluorescence of Gpd1p^WE^ hemizygote was high throughout the incubation period (**Figure 5B**, *p*-value < 0.001, Wilcoxon signed rank test). Likewise, after only 3 h of incubation the fluorescence of the WE × SA reciprocal hemizygote carrying Gpd1p^WE^ was 35% greater than that of the hemizygote carrying Gpd1p^WE^, and this trend was significant throughout the entire incubation period (**Figure 5C**, *p*-value < 0.001, Wilcoxon signed rank test). These results demonstrate the stronger expression induction profile of *pGPD1^WE^* and suggests that recessive *trans*-effects, such as post-transcriptional modifications, could negatively impact Gpd1p^WE^ expression levels, which would explain the glycerol yield differences among the *GPD1* allelic variants. We compared the coding *GPD1* sequence between strains and identified six synonymous polymorphisms. Thus, we rule out the possibility of a polymorphism that could be targeted by alternative post-translational modifications (i.e., phosphorylation or acetylation). This being said, one of the synonymous polymorphisms in the *GPD1^WE^* allele reduces the CAI respect to the other variants (*GPD1^WA^*, *GPD1^NA^* and *GPD1^SA^*); specifically, the WE allele carries the ACC codon, whereas the WA, SA and NA alleles carry an ACT codon, yet both code for threonine (T186) (**Figure 5D**).

## Discussion

Here, we explored the natural genetic variation of *S. cerevisiae* to determine how ASE can modulate glycerol production. Differences in metabolites production, such as glycerol and ethanol, were found among strains representative of the main genetic clusters recognized for this species. Interestingly, a non-wine strain had greater glycerol yields (**Figure 1B**) yet similar ethanol yields when grown in SWM. This result agrees with previous results by our group for fermentation in high nitrogen concentrations (Salinas et al., 2012). Despite this, here the wine strain yielded more glycerol than that found in the previous study, suggesting that glycerol production is influenced by nitrogen concentration. Indeed, it has been shown that the ratio of carbon to nitrogen in wine musts can significantly alter fermentation performance (Varela et al., 2004), and it is well known that strains differ in their ability to assimilate nitrogen (Crepin et al., 2012; Cubillos et al., 2017; Brice et al., 2018). During fermentation, strains can retain glycerol when under osmotic stress by decreasing the glycerol dissimilation and therefore total extracellular glycerol yields, thus impacting the final product of the fermentation (Nevoigt and Stahl, 1997; Hohmann, 2002). Yet, our results were obtained under static and small volume fermentations, and therefore we believe some differences can be expected when scaling up to larger industrial volumes.

Phenotypic differences between yeast strains can originate from polymorphic coding or non-coding regions (Thompson and Cubillos, 2017). In the analyzed strains, we found only synonymous polymorphisms in the coding portion of *GPD1*. Therefore, it is likely that polymorphisms in the regulatory region are the cause of the observed genotype – phenotype variation, however, we cannot rule out that translation speed could impact protein levels. Indeed, previous reports have demonstrated that expression variants can directly impact phenotypic differences between yeast isolates (Gerke et al., 2009; Salinas et al., 2016; Cubillos et al., 2017). In an earlier study, we have demonstrated that the four strains chosen for this study have different ASE levels (Salinas et al., 2016; Cubillos et al., 2017), and this can directly impact oenological phenotypes such as nitrogen assimilation or fermentation capacity (Salinas et al., 2016). The results presented herein of glycerol yields are consistent with this. The non-wine alleles of *ADH3* and *GPD1* in reciprocal hemizygotes (both exhibit ASE in at least a single cross involving the wine strain, **Supplementary Table S3**) produced higher glycerol levels, consumed lower amounts of sugar, and exhibited greater glycerol yields. In the case of the *ADH3^SA^* and *GPD1^SA^* alleles, we observed an antagonistic effect relative to the glycerol yields reported in parental strains (**Figures 1**, **2**). Antagonistic alleles and QTLs, refer as those alleles with a different effect from their parental origin, have been extensively described in yeast for different phenotypes and crosses (Liti and Louis, 2012) and together with other unlinked variants can expand the phenotypic landscape (Cubillos et al., 2011). These results together suggest that strains differ in their metabolic fluxes, and *cis*- and *trans*-regulation significantly impacts glycerol yields. Moreover, our results demonstrate that non-wine alleles can be potential targets of genetic improvement aimed at increasing glycerol yields. Indeed, several studies have targeted *GPD1* over-expression in wine strains to favor glycerol production. For example, introduction of a high copy number vector containing the coding portion of *GPD1* controlled by the *ADH1* promoter into the commercial wine strains K1M, VL1 and BC increases glycerol production by three-fold, while ethanol production is reduced (Cambon et al., 2006). The effective modulation of glycerol and ethanol production was affected by an increase in the production of undesirable secondary metabolites exceeding thresholds allowed for wine. Specifically, the production of acetate, acetaldehyde, and acetoin due to the redox imbalance generated by the overproduction of glycerol confers unacceptable aromas and flavors to wine. Alternatively, the overexpression of *GPD1* complemented by the overexpression of *BDH1* increases acetoin reduction to produce 2,3-butanediol, a compound that has neutral sensory properties (Ehsani et al., 2009). Yet, similar approaches should be targeted for natural variants. Here, we have identified differences in glycerol yields between *GPD1* variants and evaluated their effect in different genetic backgrounds.

Previous QTL mapping efforts have identified *GPD1* variants affecting glycerol and ethanol production, however the effect of these polymorphisms is unclear (Hubmann et al., 2013b). More difficult than generating genetically modified strains, however, is identifying and quantifying the polymorphisms within *GPD1* that underlie phenotypic differences. In this context, regulatory regions are known to finely influence phenotypes (Wray, 2007; Gerke et al., 2009; Salinas et al., 2016), and here we suggest that differences upstream the ATG start site are partly responsible for expression differences between strains. From the luciferase reporter assay we show increased expression in strains containing the wine allele. This result is in contrast with the lower glycerol yields found for reciprocal hemizygotes containing the wine alleles (**Figure 2**). Interestingly, greater expression of *GPD1^WE^* was found in parental strains and reciprocal hemizygotes, suggesting a robust *cis* effect (**Figures 3**, **4**). Indeed, our results agree with other reports in model organisms demonstrating that *cis*-variants explain a large proportion of expression differences between alleles (Brem et al., 2002; Yvert et al., 2003; Kliebenstein, 2009; McManus et al., 2010; Goncalves et al., 2012; Cubillos et al., 2014; Thompson and Cubillos, 2017), however, *trans*-eQTLs impact the expression of a greater number of genes (Brem et al., 2002; Yvert et al., 2003). Remarkably, we observed that *GPD1* was only expressed in strains grown in fermentation conditions and not in laboratory settings. The wine strain responded positively to fermentation and activation of the *GPD1* promoter was high; the mRNA levels of this strain exceeded those of other strains (**Figure 3**).

The comparison of allele expression allowed us to identify at least three different *GPD1* regulatory variants (**Figure 3**). The existence of these unique variants indicates that fine-tuning gene expression utilizing natural variants is possible. While significant differences in expression among variants were evident from the gene expression profiles (**Figure 3**), these expression patterns did not fully reflect the relative glycerol yield differences when the alleles were introduced into other strains (**Figure 3D**). One possibility for this discrepancy is that our experimental approach was insufficient to identify mild phenotypic differences due to *cis* regulation; thus, more sensitive experiments should be conducted in the future. Also, *cis*-regulatory variants can be found up to10 kb from the targeted gene and therefore by only considering 700 bp upstream the ATG start site we might be missing variants with a stronger effect upon glycerol production (Zheng et al., 2010), yet variants with stronger effects upon gene expression and phenotypes are mostly found nearby regulated genes. A more likely hypothesis is the existence of a *trans*-factor, which would agree with the patterns observed in the allele swap experiments and the lack of a positive correlation between *GPD1* expression levels, Gpd1p and glycerol yields. From the Gpd1p-mCherry fusions we found low protein levels only in the parental WE strain and not in the reciprocal hemizygotes (**Figure 3**). This suggests that a *cis*-active module strongly induces *GPD1* mRNA expression in the WE strain however a recessive post-transcriptional *trans*-acting factor could be downregulating Gpd1p decreasing glycerol yields. One could argue that technical settings could be responsible for differences between the *pGPD1-Luc* expression patterns (estimated under 200 μL in microcultivation conditions) and glycerol yields (estimated in fermentations utilizing 12 mL), however, it has been previously demonstrated that biomass and cells physiological states under microcultivation conditions correlate with larger volumes cultures (Warringer and Blomberg, 2003) and many of these findings are relevant under wine fermentation conditions (Gutierrez et al., 2013; Ibstedt et al., 2015; Brice et al., 2018; Peltier et al., 2018) and many other environments (DeLuna et al., 2010 #1056). In this context, we found a positive correlation between our Gpd1p-mCherry fusions and glycerol yields, suggesting that both set-ups would be comparable. Indeed, our findings are in line with several studies that demonstrate that mRNA levels do not necessarily correlate with protein levels due to post-transcriptional regulation that directly impacts the phenotypic outcome. As such, low protein levels can result from accelerated mRNA degradation (Liu et al., 2016). Indeed, post-transcriptional regulation of glucose production has been demonstrated in yeast, where gluconeogenic mRNA targets, such as *FBP1* and *PCK1*, are degraded (Yin et al., 2000). Apparent *GPD1* epistatic interactions have also been observed in other similar studies (Hubmann et al., 2013b). Overall, the identification of the mechanisms regulating *GPD1* ASE is a challenge. This being said, our luciferase kinetics approach did allow us to determine expressions pattern through time. It has recently been reported that time-resolved experiments are significantly more informative than genetic perturbations for inferring metabolic adaptation (Goncalves et al., 2017); thus, we were able to profile how *GPD1* regulatory regions respond to environmental perturbations through time in different genetic backgrounds.

Based on our gene expression and protein fusions assays conducted in SWM, we show that natural *GPD1* variants produce different glycerol levels and yields. Depending on the strain, this variation in glycerol production is controlled by *cis* and/or *trans* regulators, thought the *trans*-factors involved remain to be identified. These *trans*-factors likely module mRNA degradation decreasing overall Gpd1p levels. Identification of these factors requires further approaches, such as QTL mapping or Genome wide association studies involving a large number of wine strains. Indeed, our previous findings demonstrate that *RIM15* is responsible for differences in glycerol production in a WE x SA recombinant population (Salinas et al., 2012; Kessi-Perez et al., 2016). Future studies of epistatic interactions could help to determine whether differences among strains are due to *trans*-factors. Nevertheless, the set of *GPD1* regulatory variants characterized here can be used in different strains to modulate *GPD1* expression (in fermentation conditions) and glycerol production. It remains to be explored if these observations can be applied in the wine industry under larger fermentations and real industrial settings.

## Author Contributions

FS, VG, and FC designed the experiments. ST, MC, FS, VA, VD, CB, and VR performed the experiments. ST, FS, VG, CM, LL, and FC discussed the results and experiments. FS and FC wrote the paper. CM, LL, and FC provided reagents.

## Conflict of Interest Statement

The authors declare that the research was conducted in the absence of any commercial or financial relationships that could be construed as a potential conflict of interest. The reviewer PM and handling Editor declared their shared affiliation.

## Supplementary Material

FIGURE S1 *GPD1* gene expression profiles in **(A)** parental SWM, **(B)** parental YNB, and **(C)** allele swapped strains in SWM. Raw luminescence and OD values are provided.

FIGURE S2 *GPD1* gene expression profiles in reciprocal hemizygotes. Raw luminescence and OD values are provided.

FIGURE S3 Gpd1p protein levels in parental and reciprocal hemizygote strains. Raw fluorescence and OD values are provided.

TABLE S1 Primers used in this study.

TABLE S2 Physico-chemical characteristics of must after fermentations.

TABLE S3 Genes involved in ethanol and glycerol production in yeast and evaluated for ASE.

TABLE S4 Reciprocal hemizygotes’ physico-chemical characteristics of must after fermentations.
