# Supplementary figures and images for: GPD1 and ADH3 Natural Variants Underlie Glycerol Yield Differences in Wine Fermentation
Source: Front Microbiol. 2018 Jul 3;9:1460. doi: 10.3389/fmicb.2018.01460 (PMC6037841; doi:10.3389/fmicb.2018.01460)

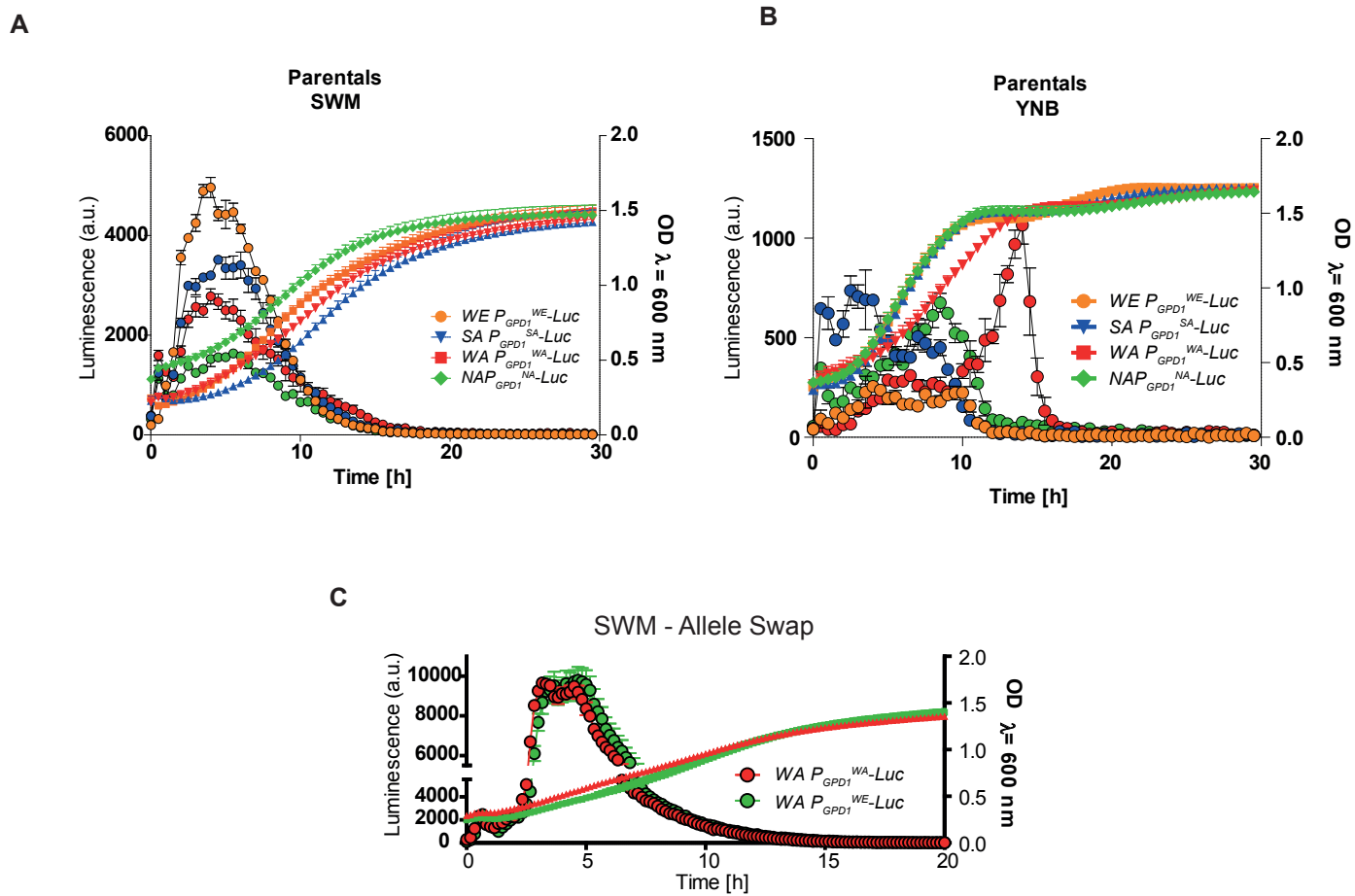

Figure S1

Supplement: FIGURE S1 — GPD1 gene expression profiles in (A) parental SWM, (B) parental YNB, and (C) allele swapped strains in SWM. Raw luminescence and OD values are provided. [file Image_1.PDF]

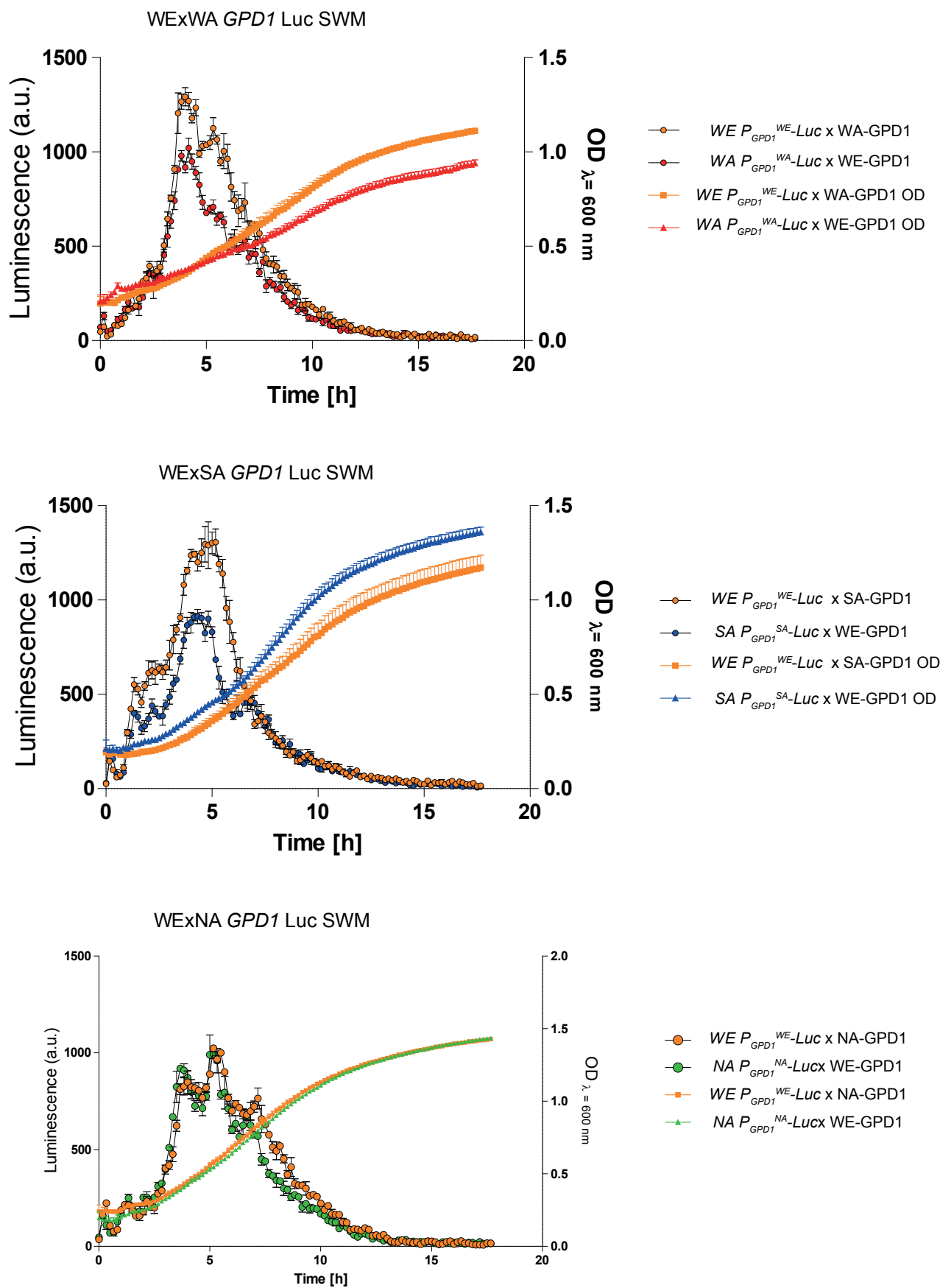

Figure S2

Supplement: FIGURE S2 — GPD1 gene expression profiles in reciprocal hemizygotes. Raw luminescence and OD values are provided. [file Image_2.PDF]

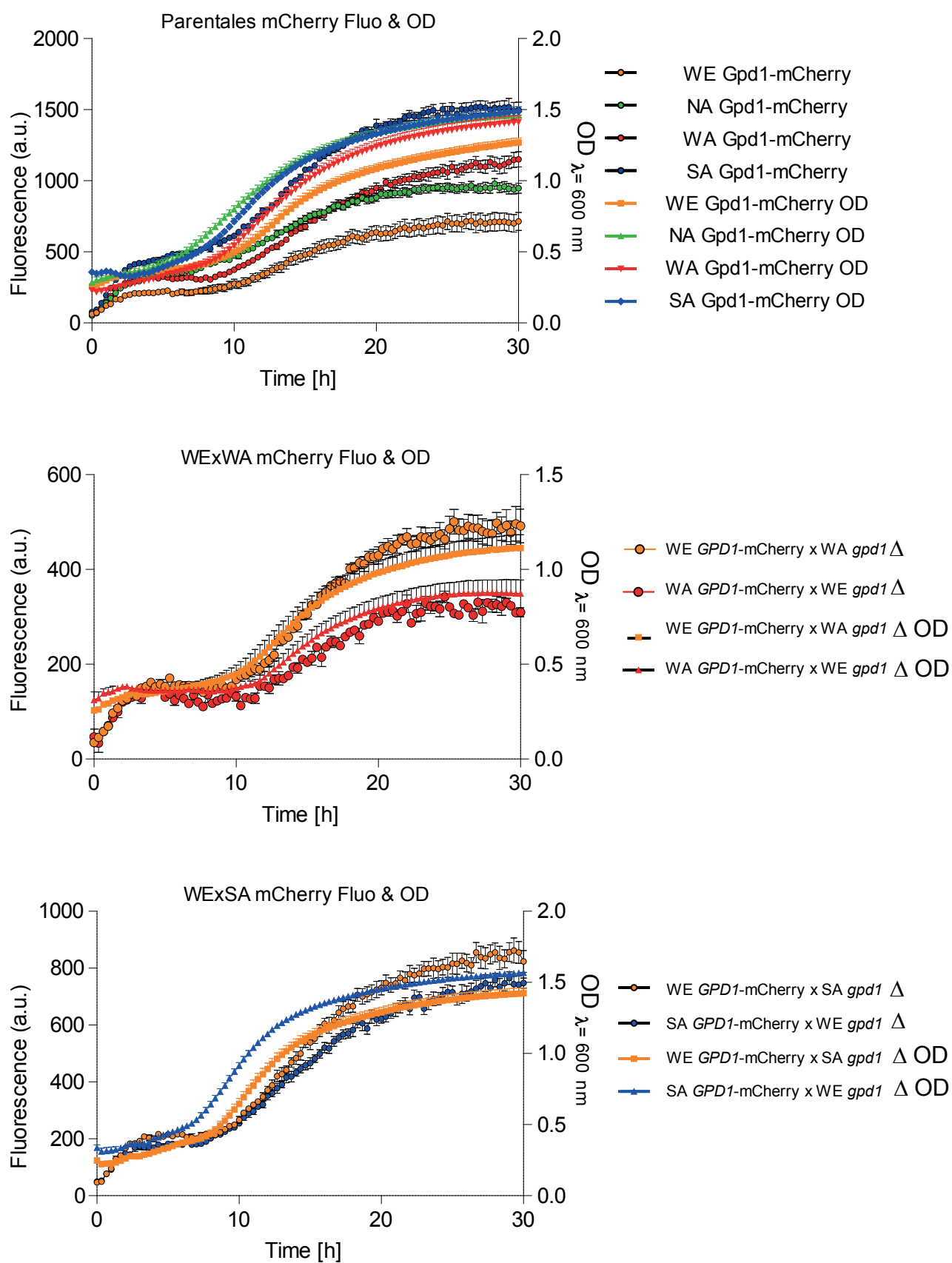

Figure S3

Supplement: FIGURE S3 — Gpd1p protein levels in parental and reciprocal hemizygote strains. Raw fluorescence and OD values are provided. [file Image_3.PDF]
